# Arsenic Methylation Capacity and Metabolic Syndrome in the 2013–2014 U.S. National Health and Nutrition Examination Survey (NHANES)

**DOI:** 10.3390/ijerph15010168

**Published:** 2018-01-22

**Authors:** Clare Pace, Julie Smith-Gagen, Jeff Angermann

**Affiliations:** 1 Department of Environmental Science, University of Nevada, Reno, NV 89557, USA; clare.pace@gmail.com; 2 School of Community Health Sciences, University of Nevada, Reno, NV 89557, USA; jsmithgagen@unr.edu

**Keywords:** arsenic, methylation, metabolic syndrome, U.S. NHANES

## Abstract

Arsenic methylation capacity is associated with metabolic syndrome and its components among highly exposed populations. However, this association has not been investigated in low to moderately exposed populations. Therefore, we investigated arsenic methylation capacity in relation to the clinical diagnosis of metabolic syndrome in a low arsenic exposure population. Additionally, we compared arsenic methylation patterns present in our sample to those of more highly exposed populations. Using logistic regression models adjusted for relevant biological and lifestyle covariates, we report no association between increased arsenic methylation and metabolic syndrome in a population in which arsenic is regulated at 10 ppb in drinking water. However, we cannot rule out the possibility of a positive association between arsenic methylation and metabolic syndrome in a subsample of women with normal body mass index (BMI). To our knowledge this is the first investigation of arsenic methylation capacity with respect to metabolic syndrome in a low exposure population. We also report that methylation patterns in our sample are similar to those found in highly exposed populations. Additionally, we report that gender and BMI significantly modify the effect of arsenic methylation on metabolic syndrome. Future studies should evaluate the effectiveness of arsenic policy enforcement on subclinical biomarkers of cardiovascular disease.

## 1. Introduction

Exposure to arsenic has been epidemiologically linked to myriad health conditions including cardiovascular disease, diabetes, and cancer [1]. Exposure to inorganic arsenic most commonly occurs through ingesting contaminated water sources and arsenic biotransformations in the liver increase the presence of highly toxic methylated arsenic metabolites [2,3,4]. Methylated arsenicals increase reactive oxygen species (ROS) to a greater extent than inorganic arsenic, thus, arsenic methylation may be associated with increased disease prevalence [3].

Arsenic is a widespread environmental contaminant commonly found in air, water, soil, and sediments. Arsenic levels in soil range from 1 to 40 mg/kg, and arsenic levels in air range from 0.007 to 28 ng/m^3^ in rural areas to 3–200 ng/m^3^ in urban areas [5,6]. Untreated waters can reach several hundred to several thousand ppb arsenic, and the World Health Organization (WHO) recommends a maximum contaminant level (MCL) for arsenic in drinking water of 10 ppb [6]. Arsenic can be inhaled, ingested, or absorbed through the skin. The American Conference of Governmental Hygienists recommend a Biological Exposure Index (BEI) level of 35 μM for total urine arsenic [7].

Arsenic has been classified as a strong carcinogen [8], and is estimated to contribute to as much as 21.4% of all causes of mortality in highly exposed regions of the world [9]. Chronic exposure to arsenic has been associated with elevated risk of cardiovascular disease (CVD) endpoints including ischemic heart disease incidence and mortality in several American Indian populations with water sources measured up to 21 ppb arsenic [10] and among populations in Bangladesh, over half of which consume ground water that substantially exceeds 50 ppb arsenic [9]. Increased rates of cerebrovascular disease prevalence have been observed among residents of Taiwan with water sources that can exceed 300 ppb arsenic [11], and increased severity of atherosclerosis was observed among subjects in rural Taiwan with median arsenic concentrations in drinking water between 700 and 930 ppb [12]. Additionally, a recent meta-analysis demonstrated a positive association between arsenic exposure and hypertension among 11 cross-sectional studies of subjects with various levels of arsenic exposure [13].

Arsenic exposure is also associated with a diagnosis of metabolic syndrome, a risk factor for CVD. Metabolic syndrome is defined as the presence of at least three out of five components including elevated fasting glucose, hypertension, elevated triglycerides, large waist circumference, and low HDL cholesterol [14]. Inorganic arsenic exposure was associated with a clinical diagnosis of metabolic syndrome, elevated plasma glucose, and elevated blood lipids among residents in an industrial region of Taiwan exposed to arsenic both occupationally and through water sources measured up to 16 ppb [15]. Importantly, inflammation and oxidative stress are significant mechanisms linking metabolic syndrome and cardiovascular disease [16]. Furthermore, both arsenic exposure and arsenic methylation are associated with oxidative stress and the generation of ROS [2,17,18], thus, there is a plausible mechanistic link between arsenic and metabolic syndrome. 

Arsenic methylation capacity is a recognized risk factor for arsenic toxicity, and may contribute to CVD susceptibility. Inorganic arsenic (iAs) undergoes biotransformation in which iAs is methylated to monomethylarsonic acid (MMA(V)). MMA(V) is then reduced to monomethylarsonous acid (MMA(III)), which is methylated to dimethylarsinic acid (DMA(V)). DMA(V) is then reduced to dimethylarsinous acid (DMA(III)). Methylated arsenic species, especially those in the trivalent form, are significantly more toxic than inorganic arsenic compounds [2,3,5]. Arsenic methylation capacity is measured as the proportion of methylated arsenicals in urine or as a ratio of MMA:iAs (termed primary methylation index or PMI) and ratio of DMA:MMA (termed secondary methylation index or SMI). Arsenic methylation facilitates urinary excretion, yet also increases the bioavailability of toxic methylated arsenicals [3]. Interestingly, methylation capacity is not highly associated with inorganic arsenic exposure [19], but is influenced by genetics, gender, age, and BMI [20,21]. 

Incomplete arsenic methylation is characterized by a pattern of higher urine proportion MMA, lower urine proportion DMA, increased PMI, and decreased SMI, whereas more complete arsenic methylation is characterized by a pattern of lower urine proportion MMA, higher urine proportion DMA, decreased PMI and increased SMI. Although only a limited number of studies have investigated the impact of arsenic methylation on cardiovascular disease or metabolic syndrome, the available evidence suggests that complete methylation may be a contributing factor [22]. Importantly, this association has not been investigated in a relatively low arsenic-exposed population. Indeed, reports of adverse health outcomes associated with arsenic exposure and arsenic methylation have originated from regions where arsenic concentrations are unregulated or significantly under-regulated and thus exceed current safety guidelines. In contrast, we assessed the relationship between arsenic methylation and pre-clinical biomarkers of cardiovascular disease in a general sample of the U.S. where despite widespread compliance with current treatment standards for arsenic in food and water, appreciable concentrations of arsenic are still detectable in the blood and urine of U.S. residents [23,24,25,26]. 

The present study uses U.S. NHANES 2013–2014 data to investigate the association between urine %iAs, %MMA, %DMA, and indices of primary methylation (PMI) and secondary methylation (SMI), with respect to a clinical diagnosis of metabolic syndrome. 

## 2. Materials and Methods

### 2.1. Study Population 

Data for this study were acquired from the U.S. National Health and Nutrition Examination Survey (NHANES) website [27]. Complete protocols for sample selection are described elsewhere [28]. In brief, NHANES is a national, population-based, cross-sectional study representing the civilian, non-institutionalized U.S. population. Demographic information is collected via survey and a physical examination is performed on all subjects. Biological samples are collected from nationally representative subsamples. The present study draws from subjects selected for the environmental subsample containing urinary arsenic concentrations which comprises approximately one third of the total U.S. NHANES population, and subjects selected to provide blood samples in the morning fasting session, comprising approximately half of the total U.S. NHANES population. Both datasets were entered into SPSS (IBM Corp. IBM SPSS Statistics for Macintosh, Version 23.0. Armonk, NY, USA) and joined based on subject ID number. Subjects selected for both subsamples were included. 

### 2.2. Exclusion Factors 

Pregnancy was an exclusion factor in the present study based on the influence of pregnancy on several measures used to assess metabolic syndrome (e.g., waist circumference, blood pressure, and cholesterol measures). Pregnancy status was determined by NHANES personnel using the Icon 25 hCG test kit (Beckman Coulter) administered to women age 20–44. In the present study, we excluded subjects under the age of 20 because arsenic metabolism is believed to differ between youth/adolescents and adults [29] and metabolic syndrome is rare among subjects under 20. We also excluded subjects who were missing data on metabolic syndrome components and covariates (age, gender, race/ethnicity, smoking status, poverty income ratio (PIR), urine creatinine, urine arsenobetaine, or BMI). Our final analytical sample contained 957 subjects. 

### 2.3. Speciated Arsenicals 

Concentrations of iAs(III), iAs(V), (MMA(III + V)), and (DMA(III + V)) were determined by NHANES personnel using high performance liquid chromatography (HPLC) to separate the species coupled to Multi-Element Inductively Coupled Plasma-Mass Spectrometry (ICP-DRC-MS) [30]. The lower limits of detection (LLOD) were 0.12 μg/L (iAs(III)), 0.79 μg/L (iAs(V)), 0.20 μg/L (MMA(III+V)) and 1.91 μg/L (DMA(III+V)). For our analysis, subjects with arsenic species below detection were assigned a concentration of the LLOD/√2, representing 29.8%, 24.5%, and 26.9%, for iAs(III), DMA, and MMA, respectively. We eliminated iAs(V) from subsequent analysis as 98.3% of samples were below detection limits for this measure. In the present study, MMA and DMA values are reported with combined valence states III and V, as methylated arsenic species’ values were not specified in the available NHANES dataset. Total arsenic concentration, % arsenic species, and indices of methylation were calculated for the present study using the following formulas:

Total Arsenic = iAs + MMA + DMA

%iAs = ((iAs/total arsenic) × 100)

%MMA = ((MMA/total arsenic) × 100)

%DMA = ((DMA/total arsenic) × 100)

PMI = MMA/iAs

SMI = DMA/MMA

PMI and SMI were right skewed and log adjusted for all subsequent analyses. Urinary %iAs, %MMA, and %DMA were right skewed but non-log normal, thus untransformed values were used in subsequent analyses. Non-log adjusted values are presented for descriptive purposes. 

### 2.4. Metabolic Syndrome Components 

Physical and biological data pertaining to metabolic syndrome were obtained from the NHANES website [28]. In the present study, a diagnosis of metabolic syndrome was given to subjects with at least three of the following conditions; fasting glucose ≥100 or medication taken to control glucose; systolic or diastolic blood pressure ≥130/85 mmHg or medication taken to control blood pressure; triglycerides ≥150 mg/dL; waist circumference ≥102 cm (males) or ≥88 cm (females); and HDL-cholesterol <40 mg/dL (males) or <50 mg/dL (females) [14]. 

### 2.5. Fasting Glucose 

Glucose concentrations were determined by NHANES personnel using the Beckman Unicel DxC800 Synchron method. The complete protocol is published elsewhere [30]. In brief, oxygen electron circuits determine the rate of oxygen consumption, which is directly proportional to the concentration of glucose in the sample. In the present study, subjects were classified as having elevated fasting glucose if their fasting serum glucose reached or exceeded 100 mg/dL, if they reported currently taking insulin, or if they reported currently taking a diabetic pill. 

### 2.6. Hypertension

After resting in a seated position for 5 min, three or four consecutive blood pressure measurements were taken by NHANES personnel. In the present study we eliminated the first measurement and averaged the remaining blood pressure measurements in accordance with recommendations from the American Heart Association council on High Blood Pressure Research [31]. Subjects whose average blood pressure reached or exceeded 130/85 mmHg were considered to have elevated blood pressure. Subjects currently taking blood pressure lowering medication were also classified in the high blood pressure group, as determined by questionnaire administered by NHANES personnel. 

### 2.7. Elevated Triglycerides 

Triglycerides were measured by NHANES personnel in refrigerated serum using the Beckman Unicel DxC800 Synchron timed-endpoint method [30]. Subjects were classified as having elevated triglycerides if their serum triglycerides reached or exceeded 150 mg/dL. 

### 2.8. Waist Circumference

Waist circumference measurement was determined by NHANES trained health technicians and was measured at the high point of the iliac crest at minimal respiration to the nearest 0.1 cm [32]. Men and women were classified with ‘large waist circumference’ if their waist circumference reached or exceeded 102 cm and 88 cm, respectively. 

### 2.9. Low HDL Cholesterol

The Roche Cobas 6000 chemistry analyzer method was used by NHANES personnel to measure serum cholesterol [30]. In brief, this endpoint reaction measures HDL-cholesterol using an ion specific electrode (ISE) and photometric measuring system. A designation of low HDL cholesterol was assigned to men with HDL cholesterol below 40 mg/dL and women with HDL cholesterol below 50 mg/dL. 

### 2.10. Covariates

Age, gender, race/ethnicity and smoking status were determined by questionnaire during household interviews administered by trained and NHANES certified personnel [28]. In the present study, smokers were defined as subjects who responded in the affirmative to smoking cigarettes ‘every day’ or on ‘some days’. A measure of creatinine concentration was determined by NHANES personnel for each urine sample via the enzymatic method using the Roche/Hitachi Cobas 6000 Analyzer (Roche Diagnostics, Indianapolis, IN, USA) [30], and was considered as an independent variable [33]. Arsenobetaine, a measure of arsenic found in seafood that may confound measures of total urinary arsenic, was measured by NHANES personnel via HPLC -ICP-DRC-MS [30]. Poverty income ratio (PIR) was calculated by NHANES personnel according to the Department of Health and Human Service (HHS) guidelines by dividing family income by the poverty guidelines specific to the survey year [30]. BMI measurements were collected by trained NHANES health technicians and are expressed as weight in kilograms divided by height in meters squared rounded to one decimal place. For the present study, BMI was categorized as normal (BMI < 25.0), overweight (25.0 ≤ BMI < 30.0), or obese (BMI ≥ 30.0). Complete protocols for all NHANES data collection methods are published elsewhere [30].

### 2.11. Statistical Analysis 

Descriptive and statistical analyses were conducted with SPSS v23 (IBM Corporation, Armonk, NY, USA). When combining data from more than one sample, using the sampling weight of either subsample is not recommended by NHANES as it could result in unreliable estimates [34]. Thus, the complex sampling design was ignored because the study population belonged to two subsamples (environmental subsample and fasting subsample). We used logistic regression to assess the relationships between urine %iAs, urine %MMA, urine %DMA, PMI, and SMI with the dependent variable metabolic syndrome, defined as subjects with 3 or more of the following components: fasting glucose ≥100 or medication taken to control glucose; systolic or diastolic blood pressure ≥130/85 mmHg or medication taken to control blood pressure; triglycerides ≥150 mg/dL; waist circumference ≥102 cm (males) or ≥88 cm (females); and HDL-cholesterol <40 mg/dL (males) or <50 mg/dL (females) [14]. Covariates were evaluated in the literature and statistically using Mann-Whitney-U test, Kruskal Wallace test, χ^2^ test, or student-*t* test, and statistically relevant or biologically justified covariates were included in the final adjusted model. We investigated plausible interactions and stratified our models by BMI and gender. We considered using BMI as a dependent variable but based on statistical collinearity between metabolic syndrome and BMI, we selected metabolic syndrome as our dependent variable and stratified by BMI category. A sensitivity analysis was conducted among subjects who exceeded baseline measures for arsenic species (iAs *n* = 635, MMA *n* = 700, DMA *n* = 732, PMI *n* = 585, SMI *n* = 681), and a subgroup analysis was conducted on non-diabetic subjects (*n* = 871). We also considered the effect of controlling for total arsenic and/or arsenobetaine, and the inclusion of urinary creatinine as an independent variable in our main models. Urinary creatinine was considered as an independent variable rather than a covariate because adjusting for creatinine has been suggested to confound the relationship between total urinary arsenic and arsenic metabolism [33].

## 3. Results

### 3.1. Study Population & Metabolic Syndrome

Table 1 provides study participant characteristics. The mean age of the study population was 47 years. Approximately 51% of subjects were male and 46% of subjects were white. Three hundred and twenty six subjects (34.1%) met the clinical diagnostic criteria for elevated fasting glucose, 425 subjects (44.4%) had elevated blood pressure or were taking medication to lower blood pressure, 261 subjects (27.3%) had elevated triglycerides, 526 subjects (55.0%) had large waist circumference, and 288 subjects (30.1%) had low HDL cholesterol. Overall, 331 (34.6%) of subjects met the inclusion criteria for metabolic syndrome. Our sample had 9.68% (±6.70), 11.69% (±9.94), and 78.63% (±9.94) urine iAs, MMA, and DMA, respectively (Table 1). Urine percent MMA ranged from below detection limits to 34.22%, and urine percent DMA ranged from 41.16% to 98.74%. The average total urinary arsenic in our sample of the U.S. NHANES 2013–2014 population was 15.67 μg/L.

### 3.2. Covariate Association with Arsenic Measures & Metabolic Syndrome

We were interested in the relationship between biologically relevant, literature-justified covariates and our independent and dependent variables. We first examined the association between continuous and categorical covariates with measures of arsenic methylation (Table 2). Gender was significantly associated with all measures of arsenic methylation (*p* < 0.05). Women had significantly lower %iAs and %MMA, significantly higher %DMA, and significantly increased PMI and SMI compared to men. Race/ethnicity, BMI, smoking status and PIR were associated with at least one arsenic measure (Table 2). White subjects had significantly higher %MMA compared to Black and Asian subjects, White subjects had significantly lower %DMA compared to Black subjects, and White subjects had significantly higher PMI compared to Mexican-American and Black subjects. 

Smokers had a significantly higher PMI compared to non-smokers, and subjects with a PIR < 1 had a significantly higher SMI compared to subjects with PIR ≥ 1. Subjects with obese BMI demonstrated a pattern of increased arsenic methylation: significantly lower %iAs and %MMA, and significantly higher %DMA and SMI compared to subjects with normal BMI. Age was significantly associated with all measures of arsenic methylation and urine arsenobetaine was significantly associated with %iAs, %MMA, and SMI (Table 2). These data suggest that when considered independently, female gender, non-White race, poverty, obesity, increased age, increased seafood consumption (measured as arsenobetaine), and dehydration (measured as urine creatinine) were associated with increased arsenic methylation. Next we assessed the association between covariates and our dependent variable (metabolic syndrome). Clinical diagnostic cutoff levels differ for men and women, therefore, we stratified our sample by gender. Age, BMI, smoking status, and PIR were significantly associated with metabolic syndrome in both men and women, whereas urinary creatinine and race/ethnicity were only significantly associated with metabolic syndrome in women (Table 3).

### 3.3. Logistic Regression

Our crude analysis suggested that in men, decreased urine %MMA, increased urine %DMA, and increased SMI were significantly associated with increased odds of metabolic syndrome whereas in women, decreased %iAs, increased %DMA and increased SMI were significantly associated with increased odds of metabolic syndrome (data not shown). 

Next we adjusted our gender stratified models for statistically relevant and literature justified covariates [35]. We adjusted gender-stratified models for age (continuous), race (White vs. non-White), poverty status (PIR < 1 vs. PIR ≥ 1), and smoking status (dichotomous) (Table 4). In the gender stratified, adjusted model, decreased %MMA was significantly associated with increased odds of metabolic syndrome in men, whereas increased SMI was significantly associated with increased odds of metabolic syndrome in women. 

Based on the significant overlap between subjects with metabolic syndrome and overweight/obese BMI [16], we stratified by BMI category rather than adjusting for BMI. (Table 5) provides the fully adjusted model among male subjects stratified for BMI category, and (Table 6) provides fully adjusted, BMI stratified values for women. The data suggest that BMI confounds the association between arsenic methylation and metabolic syndrome in men: our stratified regression model returned non-significant *p*-values for all associations of arsenic methylation and metabolic syndrome in men. In contrast, the data suggest that BMI modifies the effect of arsenic methylation on metabolic syndrome in women: reduced urine %MMA and increased SMI were significantly associated with metabolic syndrome in women with normal BMI, whereas the opposite trend occurred in women with overweight BMI (significantly increased %MMA and reduced SMI), and there was no significant association between arsenic methylation and metabolic syndrome in obese women. On the surface, this appears to suggest that BMI category modifies the association between arsenic methylation and metabolic syndrome, as women in each BMI category demonstrated a different relationship between methylation capacity and metabolic syndrome. We wondered if this was a true effect, or if it resulted from misclassification in our exposure variable. Therefore we conducted a sensitivity analysis by eliminating all subjects below detection limits for arsenicals. (In contrast, our main model used the convention of assigning individuals below detection limits an arsenic concentration of the LLOD/√2). After this sensitivity analysis, overweight BMI was not associated with incomplete arsenic methylation, and we concluded that the significant result in our main model detected an artifact originating from misclassification. It should also be noted that the sample size for women with normal BMI and metabolic syndrome was relatively small (*n* = 16 women) and represents less than 2% of our total sample.

We next assessed the possibility of biologically plausible interactions in our gender-stratified model (age*smoking, age*PIR, and PIR*smoking) and report no significant interactions (data not shown). Controlling for total arsenic, including urinary creatinine as an independent variable in logistic regression models [33] and excluding diabetic subjects had no significant impact on our results. Interestingly, controlling for seafood consumption (measured as urine arsenobetaine), in obese women resulted in a significant association between increased SMI and increased odds of metabolic syndrome, whereas the association was not significant in the model prior to adjusting for arsenobetaine. This suggests that the role of arsenic methylation on metabolic syndrome may have been masked by seafood consumption, whereas controlling for seafood consumption revealed a true association between increased arsenic methylation (SMI) and metabolic syndrome in women (Tables S1–S4).

## 4. Discussion

Ours is the first study to investigate the association between arsenic methylation and metabolic syndrome in a population with relatively low arsenic exposure. We report no association between arsenic methylation and metabolic syndrome after adjusting for relevant covariates in the majority of a population relying on water with arsenic levels regulated at 10 ppb. However, we can not rule out the possibility of an association between increased arsenic methylation and increased odds of metabolic syndrome among a small subsample of women with normal BMI, and among women with obese BMI.

### 4.1. Oxidative Stress & Inflammation 

Metabolic syndrome is associated with a clustering of abnormalities that lead to increased cardiovascular risk. Inflammation is an important cellular mechanism that links metabolic syndrome to cardiovascular disease [16]. For example, obesity (a central component of metabolic syndrome) is associated with overexpression of tumor necrosis factor α (TNF-α), a cell signaling protein involved in systematic inflammation [36], and high fat intake contributes to increased oxidative stress and nuclear factor (NF-κB) activation, a proinflammatory signaling pathway [37]. Additionally, the inflammatory marker C-reactive protein (CRP) statistically enhances the relationship between metabolic syndrome and coronary heart disease events [38]. Furthermore, oxidative stress is believed to contribute to metabolic syndrome [39], hyperglycemia [40], dyslipidemia [41], and obesity [42]. 

Oxidative stress has also been convincingly linked with exposure to arsenic. Indeed, exposure to inorganic arsenic and its metabolites can generate ROS and free radicals, hydroxyl radicals, nitric oxide, and superoxide anion in a variety of cell lines [2,18]. Additionally, a human study conducted among a highly arsenic exposed population in China reported that arsenic-exposed subjects demonstrated higher oxidative stress compared to control subjects (measured as increased serum levels of lipid peroxide) [17]. Importantly the same study reported a significant correlation between oxidative stress and methylated arsenic metabolites [17].

Thus, there is evidence for an association between arsenic and oxidative stress, as well as evidence of a link between oxidative stress and metabolic syndrome. This association, however, may be limited to populations exposed to high levels of arsenic. Indeed, our main finding of no association between arsenic methylation and metabolic syndrome contrasts with past investigations conducted among populations with higher overall arsenic exposure. For example, in regions of Taiwan with exposure to industrial arsenic as well as water sources measured up to 16 ppb, Wang et al., (2007) reported a positive association between total arsenic and metabolic syndrome [15] and Chen et al., (2012) reported that low primary methylation (low %MMA) was associated with a higher risk for metabolic syndrome in a population exposed to median arsenic concentrations of 700–930 ppb [22]. Although a minority of findings suggest that incomplete methylation (high urinary proportion of MMA) is a stronger contributor to cardiovascular disease [43] and hypertension [44], this may result from differences in the valence state of measured arsenicals. For example, studies demonstrating elevated toxicity of complete methylation have included trivalent and pentavalent species in calculations of urinary arsenic proportion, whereas studies reporting the opposite trend measured only the less toxic pentavalent species [43] or failed to specify the valence states of measured arsenicals [44]. In the present study, we report that in general, increased arsenic methylation is not associated with increased odds of metabolic syndrome, with the possible exception of specific subgroups.

### 4.2. Increased Toxicity of Methylated Species 

**Figure 1:**
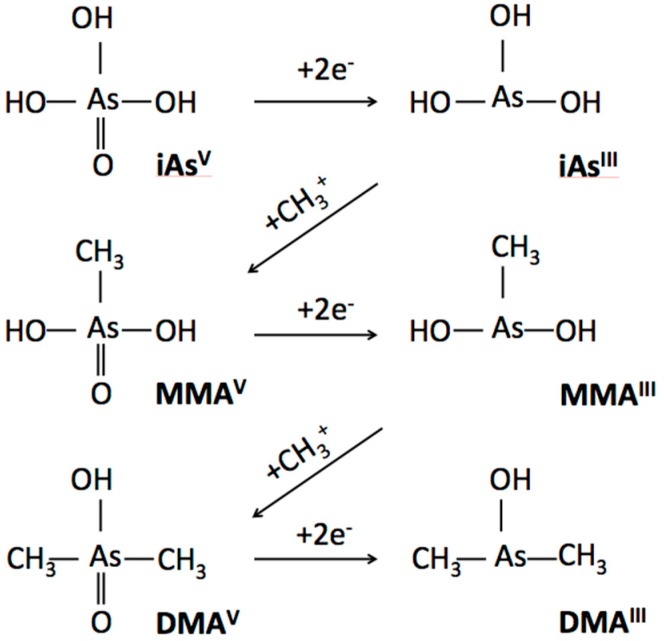
Arsenic biomethylation.

Following ingestion and tissue distribution, a series of biotransformations in the liver reduce arsenate (iAs(V)) to arsenite (iAs(III)) and arsenic methyltransferase enzymes mediate the sequential addition of methyl ions from *S*-adenosylmethionine, alternated with arsenic species’ reductions [45]. Thus, ingested inorganic arsenic is sequentially converted to its mono- and di- methylated forms and removed through the urinary system (Figure 1).

Once considered a detoxification reaction, a growing body of evidence demonstrates that arsenic methylation significantly increases toxicity and that trivalent species are more toxic than pentavalent species [2,3,5,46]. Individual variability in arsenic biotransformation is measured as the proportion of each arsenic species in urine, or by calculating PMI and SMI. Variation in urine metabolites have been associated with a variety of arsenic-related diseases [43,47] and may reflect differential bioavailability of arsenicals across individuals [48]. 

### 4.3. Arsenic Distribution Patterns

Other notable findings from the present study are the similarities in arsenic methylation patterns in our sample of the U.S. compared to regions with higher exposure, as well as similarities with respect to individual variation in methylation capacity [19]. Urinary proportions of arsenic species found in the present study are consistent with reports that most individuals have 10–30%iAs, 10–20%MMA, and 60–80%DMA, regardless of their arsenic exposure level [19]. Our sample had 9.68% (±6.70), 11.69% (±9.94), and 78.63% (±9.94) urinary iAs, MMA, and DMA, respectively. The average total urinary arsenic in the U.S. NHANES 2013–2014 population was 15.67 μg/L, considerably lower than the total urinary arsenic reported in the aforementioned studies of pre and post-clinical CVD outcomes, where total mean urinary arsenic has ranged from 53.6 μg/L among women from southwest U.S. and northwest Mexico [49] to 580 μg/L among a northern Chile population [19]. Relative consistency in average arsenic methylation capacity among different populations irrespective of arsenic exposure was also reported by Hopenhayn-Rich et al., (1996), who detected only subtle (2–3%) differences in mean urine proportion of MMA and DMA between populations exposed to inorganic arsenic at levels differing by 500 ppb [19].

In contrast to the relative consistency of arsenic methylation patterns between study populations, considerable variability has been reported in methylation capacity between individuals in the same population [35]. For example, subjects in San Pedro ranged from approximately 40% to over 90% urinary DMA and from 7% to 40% urinary MMA [19]. In the present study, urine percent MMA ranged from below detection limits to 34.22%, and urine percent DMA ranged from 41.16% to 98.74%.

### 4.4. Gender & BMI

Past studies suggest that variability in methylation capacity is due, in part to variations in genetic polymorphisms, as well as age, BMI, and gender [20,21]. Our results also demonstrate an effect of gender and BMI on arsenic metabolism. In the present study, we report that increased arsenic methylation may be associated with increased odds of metabolic disorder in gender and BMI stratified samples of the U.S. NHANES population adjusted for relevant covariates. 

Our finding that gender modifies the effect of arsenic methylation on the odds of metabolic syndrome is supported by previous research conducted by Lindberg et al., (2008) who reported that women in Bangladesh had higher methylation efficiency than men during childbearing ages (20 to 60 years) [19]. Similarly, European women between the ages of 20 and 60 had an increased rate of arsenic methylation [21]. The authors of these studies speculate that sex hormones are partially responsible for increased rates of methylation in women, as it is specific to women of childbearing age [20,21]. This is a plausible explanation for some of the observed differences in metabolic syndrome outcomes based on gender in the present study, as 79% of women in our sample were between the ages of 20–60. 

The present study also demonstrated a significant effect of BMI on arsenic methylation. Subjects with obese BMI expressed a pattern of increased arsenic methylation: significantly lower %iAs and %MMA, and significantly higher %DMA and SMI compared to subjects with normal BMI. Since increased arsenic methylation is generally associated with increased disease status, we expected to see a significant association between arsenic methylation and metabolic syndrome in obese subjects. Contrary to our expectations, we report no significant association between increased arsenic methylation and metabolic syndrome in over 98% of our sample population. We did, however, detect a positive association between increased arsenic methylation and metabolic syndrome in a small subpopulation represented by women with normal BMI.

We were interested in which clinical symptoms characterized metabolic syndrome in subjects who had metabolic syndrome and normal BMI. Among these 16 women, we found that 13 had elevated fasting glucose, 11 had elevated triglycerides, 14 had elevated blood pressure, and 12 had low HDL cholesterol. The most common combination of symptoms were elevated blood pressure and elevated glucose in combination with low HDL cholesterol (*n* = 7) or elevated triglycerides (*n* = 6). Despite the small sample size, ours is the first study to report the possibility of BMI as an effect modifier in the association between arsenic methylation and metabolic syndrome. We suggest that future studies investigate this possibility in a larger sample size.

### 4.5. Selection of Adjustment Factors 

It is worth noting that we adjusted for covariates similar to those used in other studies assessing the relationship between arsenic exposure or arsenic methylation and various aspects of metabolic syndrome. These varied somewhat based on the specific research question, but generally included age, gender, BMI, smoking status, alcohol use, diabetes, waist to hip ratio, serum lipid levels, urine creatinine, and urine arsenobetaine [10]. In the present study, we adjusted for age, poverty status, smoking status, and race, and stratified by gender and BMI category. These covariates are used in comparable studies with biological relevance verified in the literature [50]. Additionally, we report no significant effect of controlling for urine creatinine, total arsenic, or eliminating diabetic subjects. We did, however, determine that limiting our analysis to subjects above detection limits for MMA eliminated the statistical significance between incomplete arsenic methylation and metabolic syndrome in overweight subjects. This suggests that the apparent protective effect of overweight BMI was not a true effect, but rather, was due to exposure misclassification as a large number of subjects in the overweight BMI group had undetectable arsenic levels. Although we used a commonly accepted practice by assigning a value of the LLOD/√2 to subjects below detection limits, this erroneously resulted in an apparent protective effect of overweight BMI, which we deemed implausible. Sensitivity analysis revealed that this was likely an artifact of our statistical method, and not a true effect. 

We also probed our model by controlling for arsenobetaine and found that in women with obese BMI, the role of arsenic methylation on metabolic syndrome was masked by the effect of seafood consumption. By re-evaluating our final model after controlling for seafood consumption (i.e., adjusting for arsenobetaine), we detected a significant association between increased arsenic methylation (SMI) and metabolic syndrome in obese women. Navas-Acien et al., (2011) report that seafood is a significant determinant of urine concentrations of total arsenic and arsenic species [51]. Arsenobetaine is an organic form of arsenic present in high levels in seafood that is excreted unchanged via the kidneys. Navas-Acien et al., (2011) also suggest eliminating subjects who have consumed seafood in the last 24-h from statistical analyses as a further safeguard from misclassifying arsenic exposure [51]. In the present study, statistically controlling for arsenobetaine altered the statistical significance of our results among female subjects with obese BMI, whereas controlling for arsenobetaine did not alter the statistical significance of our results in women with normal or overweight BMI. We suggest that future studies should assess the possibility that the effect of seafood consumption on arsenic methylation varies as a function of BMI. 

### 4.6. Policy Implications

An important implication of our study is the relevance of public water testing and treatment. The current U.S. standard was reduced from 50 ppb to 10 ppb by the U.S. EPA’s 2001 Revised Arsenic Rule. In a recent study, Welch et al., (2017) report that arsenic levels in the general U.S. population have declined over a 12-year period in accordance with the Revised Arsenic Rule [52]. Although the current MCL fails to consider cardiovascular outcomes, diabetes, and preclinical indictors of CVD that may be associated with arsenic exposures below 10 ppb, the present study suggests that an MCL of 10 ppb arsenic is adequate to prevent arsenic exposure from contributing to metabolic syndrome in the general U.S. population. However, not all waters are subject to EPA standards, potentially placing an unequal health burden on regions with naturally higher arsenic concentrations and greater reliance on unregulated water sources. Approximately 12% of the U.S. population is served by domestic wells that are not regulated by the EPA, and 11–19% of private wells are estimated to contain arsenic in excess of 10 ppb [52,53]. For example, rural areas of the U.S. with a higher proportion of un-piped water report much higher concentrations of groundwater arsenic. A survey of 102 homes using domestic wells in rural areas of Nevada averaged 356 μg/L arsenic pre-treatment and 87 μg/L arsenic following reverse osmosis treatment [54], and arsenic concentrations were measured between 10 and 61 ppb on rural Reservation lands in Arizona [10]. Even populations using piped water on Reservation lands are at risk of consuming high concentrations of arsenic. Indeed, health-related violations relating to one or more substance above the MCL were reported among 46% of public water systems on Reservations, compared to just 7% among public water systems in the remainder of the U.S. [55]. Furthermore, socio-economically disadvantaged communities in the U.S. may face disproportionate arsenic exposure. For example, Balazs et al., (2012) report that community water systems serving a higher proportion of minority residents with low socioeconomic status in the San Joaquin Valley have higher drinking water levels and greater odds of non-compliance with current arsenic standards [56]. Unfortunately, despite the real and significant risk to rural and Native American populations, rural areas represent a small fraction of the total U.S. population and based on de-identification practices in the U.S. NHANES data, it is not possible to determine what proportion of our sample, if any, was represented by subjects living on Reservation lands. We suggest that future studies in the U.S. focus on marginalized populations who may be at greater risk of arsenic exposure due to non-compliance or lack of regulation. 

Looking beyond the U.S., global drinking water arsenic regulations range from 6 ppb in Sweden to 50 ppb in Bangladesh, though there are vast disparities in regional infrastructures that support water testing, reporting, and remediation for arsenic. For example, despite regulations, a 2009 survey of 15,000 randomized households in Bangladesh estimated that 22 million people consume water exceeding 50 ppb and 5.6 million people consume water exceeding 200 ppb arsenic [57]. However, there is no reliable estimate of the global scope of arsenic exposure due to delayed health effects, poor reporting, and low levels of awareness in some regions [6]. It is also worth noting that industrial exposures may significantly contribute to arsenic body burden. For instance, Vimercati et al., (2017) report that urinary arsenic concentrations were higher among southern Italian residents who lived close to industrial plants [58]. Therefore we suggest the need for future studies comparing the effectiveness of policy enforcing an MCL of 10 ppb arsenic on preclinical biomarkers of CVD in a model that establishes causality, as well as a need for comprehensive biomonitoring studies that consider all exposure routes. 

Important limitations in the present study include reliance on secondary data and the use of self-reported data for several measures (e.g., diabetes, smoking status, and the use of medication). All biological data was collected by U.S. NHANES personnel, and therefore we did not have access to data on methylated arsenicals speciated by valence state. Additionally, as our sample originated from two different subsamples, we were unable to use the U.S. NHANES weighting factors and are thus unable to apply our findings to the entire U.S. population. Finally, our study design allows for an interpretation of correlation but not causation. Despite these limitations, this is the first study to examine urinary arsenic methylation with respect to metabolic disorder in the U.S. We also acknowledge that our stratified sample sizes were small, and these outcomes should be assessed in a larger population. 

## 5. Conclusions

In summary, in a country where arsenic levels are regulated at 10 ppb in public drinking water, we report no significant association between arsenic methylation capacity and metabolic syndrome in the majority of our sample, however, we can not rule out the possibility of a positive association between increased arsenic methylation capacity and metabolic syndrome in women with normal BMI. Our sample represents a relatively low exposure population, and the association between arsenic methylation with metabolic syndrome should be assessed in regions of the world that lack the infrastructure for water testing and treatment and in regions of the U.S. that have a greater risk of arsenic exposure, such as American Indian Reservations, rural communities relying on untreated waters from private domestic wells, and regions with community water systems serving minority or low income populations. We emphasize the need for studies that highlight the impact of policy enforcement on urinary arsenic and health outcomes, as well as additional studies that consider the contribution of gender, BMI, and other factors that may modify the association of arsenic methylation to pre- and post-clinical cardiovascular disease status in a model that establishes causality. 

## Figures and Tables

**Table 1:** Study participant characteristics.

Characteristic	Mean (±SD) or *N* (%)	
Age (years)	47.44	(±15.55)
Sex		
Male	491	(51.30%)
Race/Ethnicity		
Non-Hispanic White	442	(46.2%)
Mexican American	120	(12.5%)
Non-Hispanic Black	204	(21.3%)
Non-Hispanic Asian	87	(9.1%)
Other Race	104	(10.8%)
PIR ^a^	2.2	(±1.61)
PIR < 1	278	(29.0%)
Smoker	196	(20.5%)
BMI ^b^ (kg/m^2^)	29.01	(±7.34)
Normal	313	(32.7%)
Overweight	290	(30.3%)
Obese	354	(36.9%)
Metabolic syndrome components		
Fasting glucose (mg/dL)	102.21	(±34.94)
Systolic blood pressure (mmHg)	122.09	(±17.53)
Diastolic blood pressure (mmHg)	68.99	(±11.55)
Serum triglycerides (mg/dL)	139.93	(±235.32)
Waist circumference (cm)	99.12	(±16.91)
HDL cholesterol (mg/dL)	52.63	(±15.28)
Urinary Arsenic Species		
iAs III ^c^ (μg/L)	0.51	(±0.45)
MMA III + V ^d^ (μg/L)	0.63	(±0.55)
DMA III + V ^e^ (μg/L)	4.68	(±5.38)
%iAs ^f^	9.68	(±6.70)
%MMA ^g^	11.69	(±5.95)
%DMA ^h^	78.63	(±9.94)
PMI ^i^	1.89	(±1.89)
SMI ^j^	10.44	(±20.72)
Number of Metabolic syndrome components		
0	179 (18.7%)	
1	233 (24.3%)	
2	214 (22.4%)	
3	193 (20.2%)	
4	104 (10.9%)	
5	34 (3.6%)	
Less than 2	626 (65.4%)	
3 or more	331 (34.6%)	

^a^ Poverty Income Ratio was calculated according to the Department of Health and Human Service (HHS) guidelines by dividing family income by the poverty guidelines specific to the survey year [30]; ^b^ Body Mass Index was calculated as weight in kilograms divided by height in meters squared rounded to one decimal place; ^c^ arsenite; ^d^ monomethylarsonous and monomethylarsonic acid; ^e^ dimethylarsinic and dimethylarsinous acid; ^f^ (iAs/(iAs + MMA + DMA) × 100); ^g^ (MMA/(iAs + MMA + DMA) × 100); ^h^ (DMA/(iAs + MMA + DMA) × 100); ^i^ (MMA/iAs); ^j^ (DMA/MMA).

**Table 2:** Distribution of arsenic measures by covariate.

Covariate	Arsenic Measures				
	Mean (SD)				
	%iAs ^a^	%MMA ^b^	%DMA ^c^	PMI ^d^	SMI ^e^
	*p*-Value ^f^	*p*-Value	*p*-Value	*p*-Value	*p*-Value
Total (*n* = 957)	9.68 (6.69)	11.69 (5.95)	78.63 (9.94)	1.89 (1.89)	10.44 (20.72)
Gender	<0.001 *	<0.001 *	<0.001 *	0.003 *	<0.001 *
Male (*n* = 491)	10.73 (6.92)	12.41 (6.02)	76.85 (10.13)	1.73 (1.67)	10.13 (27.32)
Female (*n* = 466)	8.56 (6.27)	10.93 (5.79)	80.51 (9.41)	2.05 (2.08)	10.76 (9.81)
Race/Ethnicity ^g^	0.041 *	<0.001 *	0.098	<0.001 *	0.001 *
White (*n* = 442)	9.35 (7.08)	12.53 (6.07)	78.11 (10.41)	2.18 (2.05)	9.00 (9.40)
MexAmer (*n* = 120)	10.50 (6.86)	11.30 (6.02)	78.20 (10.28)	1.38 (1.50) ^h^	9.71 (7.13)
Black (*n* = 204)	10.17 (6.52)	10.61 (5.52) ^h^	79.22 (9.24)	1.64 (1.83) ^h^	13.24 (40.59) ^h^
Asian (*n* = 87)	8.14 (4.60)	10.17 (5.64) ^h^	81.69 (8.02) ^h^	1.64 (1.42)	13.58 (16.07) ^h^
Other (*n* = 104)	10.44 (6.39)	11.96 (5.96)	77.60 (9.98)	1.71 (1.89)	9.19 (7.23)
Smoker	0.143	0.141	0.962	0.009 *	0.268
No (*n* = 761)	9.84 (6.83)	11.54 (5.90)	78.61 (10.01)	1.84 (1.87)	10.78 (22.95)
Yes (*n* = 196)	9.054 (6.11)	12.27 (6.14)	78.677 (9.72)	2.09 (1.95)	9.08 (7.13)
PIR ^i^	0.524	0.053	0.194	0.988	0.049 *
<1 (*n* = 278)	9.89 (6.57)	12.24 (5.97)	77.86 (10.16)	1.90 (1.92)	10.89 (35.18)
>1 (*n* = 679)	9.59 (6.75)	11.46 (5.93)	78.94 (9.85)	1.88 (1.88)	10.25 (9.98)
BMI	0.001 *	<0.001 *	<0.001 *	0.780	<0.001 *
Normal (*n* = 313) ^j^	10.60 (7.26)	12.72 (6.44)	76.67 (10.81)	1.85 (1.76)	9.29 (11.56)
Overwt (*n* = 290) ^k^	9.84 (6.62)	12.00 (5.51)	78.16 (9.50)	1.89 (1.73)	8.98 (7.31)
Obese (*n* = 354) ^l^	8.73 (6.10)	10.52 (5.67)	80.75 (9.09)	1.91 (2.11)	12.66 (31.52)
	**[B(Se) Beta ^m^*p*-Value ^n^]**				
Age	−0.11 (0.01) 0.268	−0.04 (0.01) −0.116	0.15 (0.02) 0.250	0.00 (0.00) 0.182	0.00 (0.00) 0.159
	0.001 *	0.002 *	0.001 *	0.001 *	0.001 *
Creatinine ^o^	0.003 (0.002) 00.03	1.59 (0.59) 0.078	−2.74 (0.97) −0.018	−0.04 (0.04) −0.037	−0.07 (0.03) −0.072
	0.233	0.011 *	0.002 *	0.220	0.008 *
Arsenobetaine ^p^	−0.026 (0.01) −0.10	−2.60 (0.29) −0.252	4.61 (0.51) 0.267	0.00 (0.02) 0.000	0.15 (0.02) 0.292
	0.003 *	0.001 *	0.001 *	0.995	0.001 *

^a^ urine % arsenite; ^b^ urine % monomethylarsonous and monomethylarsonic acid; ^c^ urine % dimethylarsinic and dimethylarsinous acid; ^d^ primary methylation index; ^e^ secondary methylation index; ^f^ *p*-values obtained from Mann-Whitney U Test comparing two groups or Kruskal-Wallis Test comparing more than two groups (* significant at (α = 0.05)); ^g^ post hoc analysis conducted with Tukey’s test; ^h^ Significant difference compared to White race, (Tukey’s post hoc test); ^i^ Poverty Income Ratio—calculated by dividing family income by the poverty guidelines specific to the survey year [30]; ^j^ BMI < 25.0; ^k^ 25.0 ≤ BMI < 30.0; ^l^ BMI ≥ 30.0; ^m^ B(Se) Beta: Unstandardized coefficient (Standard error) Standardized coefficients; ^n^ *p*-values obtained from bootstrapped linear regression (α = 0.05); ^o^ creatinine is used to normalize for hydration, ^p^ arsenobetaine is a form of arsenic found in seafood.

**Table 3:** Association between metabolic syndrome and covariates.

Covariate	Metabolic Syndrome Status					
	(No) *n* = 334	(Yes) *n* = 157	*p*-Value ^a^	(No) *n* = 292	(Yes) *n* = 174	*p*-Value
	Men			Women		
	Mean (±SD) or *n*			Mean (±SD) or *n*		
BMI			<0.001 *			<0.001 *
Normal ^b^	148	17		132	16	
Overweight ^c^	132	55		67	36	
Obese ^d^	54	85		93	122	
Smoker			<0.001 *			<0.001 *
Yes	87	42		41	26	
Non	247	115		251	148	
PIR ^e^			<0.001 *			<0.001 *
<1	38	96		85	59	
>1	238	119		207	148	
Race/Ethnicity			0.472			0.049 *
White	150	72		133	87	
MexAmer ^f^	38	22		41	19	
Black	73	38		50	43	
Asian	30	13		28	13	
Other	43	12		40	12	
Age	44.4 (±16.7)	53.6 (±15.0)	<0.001 *	44.2 (±16.3)	53.3 (±15.0)	<0.001 *
Creatinine ^g^	133.1 (±76.7)	134.1 (±73.4)	0.767	98.0 (±69.2)	109.4 (±70.1)	0.036 *
Arsenobetaine ^h^	7.9 (19.4)	6.7 (19.9)	0.266	10.4 (37.2)	6.6 (18.8)	0.477

^a^ *p*-Values obtained from 2-way χ^2^ test or one-way ANOVA for categorical variables and Mann Whitney U test for continuous covariates (* significant at (α = 0.05)); ^b^ BMI < 25.0, ^c^ 25.0 ≤ BMI < 30.0; ^d^ BMI ≥ 30.0; ^e^ Poverty Income Ratio—calculated by dividing family income by the poverty guidelines specific to the survey year [30]; ^f^ Mexican American; ^g^ creatinine is used to normalize for hydration; ^h^ arsenobetaine is a form of arsenic present in seafood.

**Table 4:** Binary logistic regression, fully adjusted ^a^.

Variable	Men			Women		
	*R*^2^	OR [95% CI]	*p*-Value ^b^	*R*^2^	OR [95% CI]	*p*-Value
%iAs ^c^	0.069	0.999 [0.970, 1.029]	0.950	0.085	0.977 [0.944, 1.012]	0.977
%MMA ^d^	0.079	0.960 [0.927, 0.994]	0.021 *	0.085	0.974 [0.940, 1.010]	0.153
%DMA ^e^	0.073	1.015 [0.995, 1.037]	0.150	0.087	1.020 [0.998, 1.044]	0.078
PMI ^f^	0.073	0.656 [0.358, 1.203]	0.173	0.081	0.956 [0.543, 1.683]	0.876
SMI ^g^	0.073	1.636 [0.834, 3.209]	0.152	0.090	2.148 [1.048, 4.402]	0.037 *

^a^ adjusted for age (continuous), poverty income ratio (PIR < 1 vs. PIR ≥ 1)—calculated by dividing family income by the poverty guidelines specific to the survey year [31], race (white vs. non-white), and smoking status (dichotomous), *R*^2^ represents Cox & Snell *R*^2^ value; ^b^ *p*-value obtained from binary logistic regression (* significant at (α = 0.05)); ^c^ urine % arsenite, ^d^ urine % monomethylarsonous and monomethylarsonic acid; ^e^ urine % dimethylarsinic and dimethylarsinous acid; ^f^ primary methylation index; ^g^ secondary methylation index.

**Table 5:** Binary logistic regression; DV: Metabolic syndrome in men, fully adjusted model ^a^.

IV	Normal BMI ^b^			Overweight BMI ^c^			Obese BMI ^d^		
	*R*^2^	OR [95% CI]	*p*-Value ^e^	*R*^2^	OR [95% CI]	*p*-Value	*R*^2^	OR [95% CI]	*p*-Value
%iAs ^f^	0.073	0.979 [0.879, 1.069]	0.639	0.101	1.017 [0.966, 1.071]	0.512	0.110	1.000 [0.946, 1.058]	0.994
%MMA ^g^	0.073	0.974 [0.888, 1.068]	0.577	0.101	1.019 [0.958, 1.083]	0.554	0.112	0.979 [0.913, 1.049]	0.549
%DMA ^h^	0.074	1.019 [0.963, 1.078]	0.519	0.102	0.986 [0.951 1.022]	0.429	0.110	1.007 [0.966 1.050]	0.728
PMI ^i^	0.072	1.179 [0.230, 6.043]	0.843	0.099	0.926 [0.312, 2.748]	0.890	0.110	0.995 [0.333, 2.979]	0.993
SMI ^j^	0.072	1.470 [0.238, 9.081]	0.678	0.106	0.477 [0.129, 1.757]	0.266	0.110	0.929 [0.275, 3.144]	0.906

^a^ adjusted for age (continuous), Poverty Income Ratio (PIR < 1 vs. PIR ≥ 1)—calculated by dividing family income by the poverty guidelines specific to the survey year [30], race (White vs. non-White), and smoking status (dichotomous); ^b^ BMI < 25.0; ^c^ 25.0 ≤ BMI < 30.0; ^d^ BMI ≥ 30.0, *R*^2^ represents Cox & Snell *R*^2^ value; ^e^ *p*-value obtained from binary logistic regression (* significant at (α = 0.05)); ^f^ urine % arsenite; ^g^ urine % monomethylarsonous and monomethylarsonic acid; ^h^ urine % dimethylarsinic and dimethylarsinous acid; ^i^ primary methylation index; ^j^ secondary methylation index.

**Table 6:** Binary logistic regression; DV: Metabolic syndrome in women, fully adjusted model ^a^.

IV	Normal BMI ^b^			Overweight BMI ^c^			Obese BMI ^d^		
	*R*^2^	OR [95% CI]	*p*-Value ^e^	*R*^2^	OR [95% CI]	*p*-Value	*R*^2^	OR [95% CI]	*p*-Value
%iAs ^f^	0.145	0.955 [0.854, 1.069]	0.426	0.179	1.026 [0.950, 1.109]	0.509	0.050	0.992 [0.942, 1.044]	0.746
%MMA ^g^	0.182	0.826 [0.701, 0.973]	0.022 *	0.219	1.113 [1.016, 1.218]	0.021 *	0.057	0.966 [0.919, 1.015]	0.174
%DMA ^h^	0.167	1.085 [0.999, 1.178]	0.053	0.203	0.951 [0.903 1.003]	0.063	0.054	1.018 [0.986 1.052]	0.273
PMI ^i^	0.160	0.644 [0.365, 1.135]	0.128	0.187	1.149 [0.912, 1.446]	0.239	0.049	0.992 [0.879 1.120]	0.896
SMI ^j^	0.175	11.485 [1.371, 96.196]	0.024 *	0.220	0.089 [0.011, 0.704]	0.022 *	0.064	2.590 [0.926, 7.243]	0.070

^a^ adjusted for age (continuous), Poverty Income Ratio (PIR < 1 vs. PIR ≥ 1)—calculated by dividing family income by the poverty guidelines specific to the survey year [30], race (White vs. non-White), and smoking status (dichotomous); ^b^ BMI < 25.0; ^c^ 25.0 ≤ BMI < 30.0; ^d^ BMI ≥ 30.0, *R*^2^ represents Cox & Snell *R*^2^ value; ^e^ *p*-value obtained from binary logistic regression (* significant at (α = 0.05)); ^f^ urine % arsenite; ^g^ urine % monomethylarsonous and monomethylarsonic acid; ^h^ urine % dimethylarsinic and dimethylarsinous acid; ^i^ primary methylation index; ^j^ secondary methylation index.

## Supplementary Materials

The following are available online at http://www.mdpi.com/1660-4601/15/1/168/s1, Table S1: Subgroup and sensitivity analyses in fully adjusted, binary logistic regression models with independent variable %MMA in women, Table S2: Subgroup and sensitivity analyses in fully adjusted, binary logistic regression models with independent variable SMI, Table S3: Subgroup and sensitivity analyses in fully adjusted, binary logistic regression models with independent variable %MMA in men, Table S4: Subgroup and sensitivity analyses in fully adjusted, binary logistic regression models with independent variable SMI.

## References

[1] Singh A.P., Goel R.K., Kaur T. (2011). Mechanisms pertaining to arsenic toxicity. Toxicol. Int..

[2] Pace C., Banerjee T.D., Welch B., Khalili R., Dagda R.K., Angermann J. (2016). Monomethylarsonous acid, but not inorganic arsenic, is a mitochondria-specific toxicant in vascular smooth muscle cells. Toxicol. In Vitro.

[3] Styblo M., Del Razo L.M., Vega L., Germolec D.R., LeCluyse E.L., Hamilton G.A., Reed W., Wang C., Cullen W.R., Thomas D.J. (2000). Comparative toxicity of trivalent and pentavalent inorganic and methylated arsenicals in rat and human cells. Arch. Toxicol..

[4] Calatayud M., Devesa V., Vélez D. (2013). Differential toxicity and gene expression in caco-2 cells exposed to arsenic species. Toxicol. Lett..

[5] Vimercati L., Carrus A., Sciannamblo G., Caputo F., Minunni V., de Nichilo G., Bellotta M.R., Gagliardi T., Bisceglia L., Assennato G. (2009). A study of factors influencing urinary arsenic excretion in exposed workers. Int. J. Environ. Health Res..

[6] World Health Organization (2001). International Programme on Chemical Safety, Arsenic and Arsenic Compounds, Environmental Health Criteria 224.

[7] American Conference of Governmental Hygienists (2007). Arsenic and Its Inorganic Compounds.

[8] Tsuda T., Babazono A., Yamamoto E., Kurumatani N., Mino Y., Ogawa T., Kishi Y., Aoyama H. (1995). Ingested arsenic and internal cancer: A historical cohort study followed for 33 years. Am. J. Epidemiol..

[9] Argos M., Kalra T., Rathouz P.J., Chen Y., Pierce B., Parvez F., Islam T., Ahmed A., Rakibuz-Zaman M., Hasan R. (2010). Arsenic exposure from drinking water, and all-cause and chronic-disease mortalities in bangladesh (heals): A prospective cohort study. Lancet.

[10] Moon K.A., Guallar E., Umans J.G., Devereux R.B., Best L.G., Francesconi K.A., Goessler W., Pollak J., Silbergeld E.K., Howard B.V. (2013). Association between exposure to low to moderate arsenic levels and incident cardiovascular disease. A prospective cohort study. Ann. Intern. Med..

[11] Chiou H.Y., Huang W.I., Su C.L., Chang S.F., Hsu Y.H., Chen C.J. (1997). Dose-Response relationship between prevalence of cerebrovascular disease and ingested inorganic arsenic. Stroke.

[12] Wang C.H., Jeng J.S., Yip P.K., Chen C.L., Hsu L.I., Hsueh Y.M., Chiou H.Y., Wu M.M., Chen C.J. (2002). Biological gradient between long-term arsenic exposure and carotid atherosclerosis. Circulation.

[13] Abhyankar L.N., Jones M.R., Guallar E., Navas-Acien A. (2012). Arsenic exposure and hypertension: A systematic review. Environ. Health Perspect..

[14] National Cholesterol Education Program (NCEP) Expert Panel on Detection, Evaluation, and Treatment of High Blood Cholesterol in Adults (Adult Treatment Panel III) (2002). Third report of the national cholesterol education program (ncep) expert panel on detection, evaluation, and treatment of high blood cholesterol in adults (adult treatment panel iii) final report. Circulation.

[15] Wang S.L., Chang F.H., Liou S.H., Wang H.J., Li W.F., Hsieh D.P. (2007). Inorganic arsenic exposure and its relation to metabolic syndrome in an industrial area of Taiwan. Environ. Int..

[16] Ritchie S.A., Connell J.M. (2007). The link between abdominal obesity, metabolic syndrome and cardiovascular disease. Nutr. Metab. Cardiovasc. Dis..

[17] Pi J., Yamauchi H., Kumagai Y., Sun G., Yoshida T., Aikawa H., Hopenhayn-Rich C., Shimojo N. (2002). Evidence for induction of oxidative stress caused by chronic exposure of Chinese residents to arsenic contained in drinking water. Environ. Health Perspect..

[18] Barchowsky A., Dudek E.J., Treadwell M.D., Wetterhahn K.E. (1996). Arsenic induces oxidant stress and nf-kappa b activation in cultured aortic endothelial cells. Free Radic. Biol. Med..

[19] Hopenhayn-Rich C., Biggs M.L., Smith A.H., Kalman D.A., Moore L.E. (1996). Methylation study of a population environmentally exposed to arsenic in drinking water. Environ. Health Perspect..

[20] Lindberg A.L., Ekström E.C., Nermell B., Rahman M., Lönnerdal B., Persson L.A., Vahter M. (2008). Gender and age differences in the metabolism of inorganic arsenic in a highly exposed population in Bangladesh. Environ. Res..

[21] Lindberg A.L., Kumar R., Goessler W., Thirumaran R., Gurzau E., Koppova K., Rudnai P., Leonardi G., Fletcher T., Vahter M. (2007). Metabolism of low-dose inorganic arsenic in a central european population: Influence of sex and genetic polymorphisms. Environ. Health Perspect..

[22] Chen J.W., Wang S.L., Wang Y.H., Sun C.W., Huang Y.L., Chen C.J., Li W.F. (2012). Arsenic methylation, gsto1 polymorphisms, and metabolic syndrome in an arseniasis endemic area of southwestern Taiwan. Chemosphere.

[23] Caldwell K.L., Jones R.L., Verdon C.P., Jarrett J.M., Caudill S.P., Osterloh J.D. (2009). Levels of urinary total and speciated arsenic in the US population: National health and nutrition examination survey 2003–2004. J. Expo. Sci. Environ. Epidemiol..

[24] Shiue I. (2014). Higher urinary heavy metal, phthalate, and arsenic but not parabens concentrations in people with high blood pressure, U.S. NHANES, 2011–2012. Int J. Environ. Res. Public Health.

[25] Shiue I., Hristova K. (2014). Higher urinary heavy metal, phthalate and arsenic concentrations accounted for 3–19% of the population attributable risk for high blood pressure: U.S. NHANES, 2009–2012. Hypertens. Res..

[26] Shiue I. (2014). Higher urinary heavy metal, arsenic, and phthalate concentrations in people with high blood pressure: U.S. NHANES, 2009–2010. Blood Press.

[27] NHANES 2013–2014 Questionnaire Data. https://wwwn.cdc.gov/nchs/nhanes/search/datapage.aspx?Component=Questionnaire&CycleBeginYear=2013.

[28] NHANES 2013–2014 Questionnaires, Datasets, and Related Documentation. https://wwwn.cdc.gov/nchs/nhanes/ContinuousNhanes/Default.aspx?BeginYear=2013.

[29] Concha G., Vogler G., Lezcano D., Nermell B., Vahter M. (1998). Exposure to inorganic arsenic metabolites during early human development. Toxicol. Sci..

[30] NHANES 2013–2014 Lab Methods. https://wwwn.cdc.gov/nchs/nhanes/continuousnhanes/labmethods.aspx?BeginYear=2013.

[31] Pickering T.G., Hall J.E., Appel L.J., Falkner B.E., Graves J., Hill M.N., Jones D.W., Kurtz T., Sheps S.G., Roccella E.J. (2005). Recommendations for blood pressure measurement in humans and experimental animals: Part 1: Blood pressure measurement in humans: A statement for professionals from the subcommittee of professional and public education of the american heart association council on high blood pressure research. Hypertension.

[32] NHANES 2013–2014 Survey Operations Manuals. https://wwwn.cdc.gov/nchs/nhanes/ContinuousNhanes/manuals.aspx?BeginYear=2013.

[33] Gamble M.V., Liu X. (2005). Urinary creatinine and arsenic metabolism. Environ. Health Perspect..

[34] Trasande L., Spanier A.J., Sathyanarayana S., Attina T.M., Blustein J. (2013). Urinary phthalates and increased insulin resistance in adolescents. Pediatrics.

[35] Vahter M. (2000). Genetic polymorphism in the biotransformation of inorganic arsenic and its role in toxicity. Toxicol. Lett..

[36] Hotamisligil G.S., Shargill N.S., Spiegelman B.M. (1993). Adipose expression of tumor necrosis factor-alpha: Direct role in obesity-linked insulin resistance. Science.

[37] Aljada A., Mohanty P., Ghanim H., Abdo T., Tripathy D., Chaudhuri A., Dandona P. (2004). Increase in intranuclear nuclear factor kappab and decrease in inhibitor kappab in mononuclear cells after a mixed meal: Evidence for a proinflammatory effect. Am. J. Clin. Nutr..

[38] Diamant M., Lamb H.J., van de Ree M.A., Endert E.L., Groeneveld Y., Bots M.L., Kostense P.J., Radder J.K. (2005). The association between abdominal visceral fat and carotid stiffness is mediated by circulating inflammatory markers in uncomplicated type 2 diabetes. J. Clin. Endocrinol. Metab..

[39] Hopps E., Noto D., Caimi G., Averna M.R. (2010). A novel component of the metabolic syndrome: The oxidative stress. Nutr. Metab. Cardiovasc. Dis..

[40] Andreeva-Gateva P., Popova D., Orbetsova V. (2001). Antioxidant parameters in metabolic syndrome—A dynamic evaluation during oral glucose tolerance test. Vutr Boles.

[41] Maeda K., Yasunari K., Sato E.F., Inoue M. (2005). Enhanced oxidative stress in neutrophils from hyperlipidemic guinea pig. Atherosclerosis.

[42] Roberts C.K., Barnard R.J., Sindhu R.K., Jurczak M., Ehdaie A., Vaziri N.D. (2006). Oxidative stress and dysregulation of NAD(P)H oxidase and antioxidant enzymes in diet-induced metabolic syndrome. Metabolism.

[43] Tseng C.H., Huang Y.K., Huang Y.L., Chung C.J., Yang M.H., Chen C.J., Hsueh Y.M. (2005). Arsenic exposure, urinary arsenic speciation, and peripheral vascular disease in blackfoot disease-hyperendemic villages in Taiwan. Toxicol. Appl. Pharmacol..

[44] Li X., Li B., Xi S., Zheng Q., Lv X., Sun G. (2013). Prolonged environmental exposure of arsenic through drinking water on the risk of hypertension and type 2 diabetes. Environ. Sci. Pollut. Res. Int..

[45] Mandal B.K., Suzuki K.T. (2002). Arsenic round the world: A review. Talanta.

[46] Vega L., Styblo M., Patterson R., Cullen W., Wang C., Germolec D. (2001). Differential effects of trivalent and pentavalent arsenicals on cell proliferation and cytokine secretion in normal human epidermal keratinocytes. Toxicol. Appl. Pharmacol..

[47] Huang Y.K., Tseng C.H., Huang Y.L., Yang M.H., Chen C.J., Hsueh Y.M. (2007). Arsenic methylation capability and hypertension risk in subjects living in arseniasis-hyperendemic areas in southwestern Taiwan. Toxicol. Appl. Pharmacol..

[48] Vahter M., Concha G. (2001). Role of metabolism in arsenic toxicity. Pharmacol. Toxicol..

[49] Gomez-Rubio P., Roberge J., Arendell L., Harris R.B., O’Rourke M.K., Chen Z., Cantu-Soto E., Meza-Montenegro M.M., Billheimer D., Lu Z. (2011). Association between body mass index and arsenic methylation efficiency in adult women from southwest U.S. and northwest Mexico. Toxicol. Appl. Pharmacol..

[50] Park Y.W., Zhu S., Palaniappan L., Heshka S., Carnethon M.R., Heymsfield S.B. (2003). The metabolic syndrome: Prevalence and associated risk factor findings in the US population from the third national health and nutrition examination survey, 1988–1994. Arch. Intern. Med..

[51] Navas-Acien A., Francesconi K.A., Silbergeld E.K., Guallar E. (2011). Seafood intake and urine concentrations of total arsenic, dimethylarsinate and arsenobetaine in the US population. Environ. Res..

[52] Welch B., Smit E., Cardenas A., Hystad P., Kile M.L. (2017). Trends in urinary arsenic among the U.S. Population by drinking water source: Results from the national health and nutritional examinations survey 2003–2014. Environ. Res..

[53] Kumar A., Adak P., Gurian P.L., Lockwood J.R. (2010). Arsenic exposure in US public and domestic drinking water supplies: A comparative risk assessment. J. Expo. Sci. Environ. Epidemiol..

[54] George C.M., Smith A.H., Kalman D.A., Steinmaus C.M. (2006). Reverse osmosis filter use and high arsenic levels in private well water. Arch. Environ. Occup. Health.

[55] National Public Water Systems Compliance Report. https://www.epa.gov/sites/production/files/2014-04/documents/sdwacom2011.pdf.

[56] Balazs C.L., Morello-Frosch R., Hubbard A.E., Ray I. (2012). Environmental justice implications of arsenic contamination in California’s San Joaquin Valley: A cross-sectional, cluster-design examining exposure and compliance in community drinking water systems. Environ. Health.

[57] Flanagan S.V., Johnston R.B., Zheng Y. (2012). Arsenic in tube well water in bangladesh: Health and economic impacts and implications for arsenic mitigation. Bull. World Health Organ..

[58] Vimercati L., Gatti M.F., Gagliardi T., Cuccaro F., De Maria L., Caputi A., Quarato M., Baldassarre A. (2017). Environmental exposure to arsenic and chromium in an industrial area. Environ. Sci. Pollut. Res. Int..

