# Supplementary material for: Arsenic Methylation Capacity and Metabolic Syndrome in the 2013–2014 U.S. National Health and Nutrition Examination Survey (NHANES)
Source: Int J Environ Res Public Health. 2018 Jan 22;15(1):168. doi: 10.3390/ijerph15010168 (PMC5800267; doi:10.3390/ijerph15010168)
Supplement: Supplementary file 1 [file ijerph-15-00168-s001.pdf]

Table S1

Subgroup and sensitivity analyses in fully adjusted[a] binary logistic regression model of women

IV: %MMA[b]

DV: Metabolic Syndrome

	Normal BMI[c]			Overweight BMI[d]			Obese BMI[e]		
**Additional Criteria**	**R^2**	**OR [95% CI]**	***p*-value[f]**	**R^2**	**OR [95% CI]**	***p*-value**	**R^2**	**OR [95% CI]**	***p*-value**
Exceeding baseline for MMA[g]	0.177	0.747 [0.588, 0.950]	0.017*	0.346	1.060 [0.947, 1.186]	0.311	0.057	0.976 [0.918, 1.037]	0.427
Non diabetic subjects	0.182	0.826 [0.701, 0.973]	0.022*	0.291	1.113 [1.016, 1.218]	0.021*	0.056	0.976 [0.928, 1.026]	0.340
Adjusted for total arsenic	0.198	0.817 [0.689, 0.968]	0.020*	0.221	1.111 [1.014, 1.216]	0.024*	0.057	0.966 [10.920, 1.016]	0.177
Adjusted for arsenobetaine[h]	0.187	0.817 [0.690, 0.970]	0.021*	0.231	1.102 [1.000, 1.210]	0.040*	0.075	0.960 [0.913, 1.009]	0.109
Adjusted for creatinine[i]	0.182	0.825 [0.699, 0.973]	0.002*	0.220	1.112 [1.015, 1.218]	0.022*	0.067	0.965 [0.917, 1.014]	0.161

[a] adjusted for age (continuous), gender, race (dichotomous, White vs. non-White) smoking status (dichotomous), and poverty status (PIR<1 vs. PIR≥1); [b] Urine % MMA; [c] BMI<25.0; [d] 25.0 ≥BMI<30.0; [e] BMI≥30.0; R^2 represents Cox & Snell R^2 value; [f] *p*-value obtained from binary logistic regression (* significant at ($\alpha = 0.05$)); [g] urine MMA ≥0.20μg/L; [h] arsenic in seafood; [i] measure of hydration

Table S2

Subgroup and sensitivity analyses in fully adjusted[a] binary logistic regression model of women

IV: SMI[b]

DV: Metabolic Syndrome

	Normal BMI[c]			Overweight BMI[d]			Obese BMI[e]		
**Additional Criteria**	**R^2**	**OR [95% CI]**	***p*-value[f]**	**R^2**	**OR [95% CI]**	***p*-value**	**R^2**	**OR [95% CI]**	***p*-value**
Exceeding baseline for MMA and DMA[f]	0.220	8.309 [1.065, 64.850]	0.043*	0.306	0.223 [0.022, 2.797]	0.207	0.050	2.290 [0.707, 7.417]	0.167
Non diabetic subjects	0.175	11.485 [1.371, 96.196]	0.022*	0.220	0.089 [0.011, 0.074]	0.022*	0.061	2.124 [0.738, 6.110]	0.162
Adjusted for total arsenic	0.188	12.244 [1.324, 113.201]	0.027*	0.221	0.093 [0.012, 0.739]	0.025*	0.064	2.580 [0.092, 7.230]	0.072
Adjusted for arsenobetaine[g]	0.180	12.550 [1.440, 109.76]	0.022*	0.229	0.119 [0.014, 0.999]	0.050	0.083	3.115 [1.080, 8.960]	0.035*
Adjusted for creatinine[h]	0.175	11.635 [1.370, 98.791]	0.025*	0.220	0.090 [0.011, 0.715]	0.023*	0.073	2.663 [0.938, 7.560]	0.167

[a] adjusted for age (continuous), gender, race (dichotomous, White vs. non-White) smoking status (dichotomous), and poverty status (PIR<1 vs. PIR≥1); [b] Secondary methylation index; [c] BMI<25.0; [d] 25.0 ≥BMI<30.0; [e] BMI≥30.0; R^2 represents Cox & Snell R^2 value; [f] *p*-value obtained from binary logistic regression (* significant at (α = 0.05)); [g] urine DMA ≥1.91µg/L, urine MMA ≥0.20µg/L; [h] arsenic in seafood; [i] measure of hydration

Table S3

Subgroup and sensitivity analyses in fully adjusted[a] binary logistic regression model of men

IV: %MMA[b]

DV: Metabolic Syndrome

	Normal BMI[c]			Overweight BMI[d]			Obese BMI[e]		
**Additional Criteria**	**R^2**	**OR [95% CI]**	***p*-value[f]**	**R^2**	**OR [95% CI]**	***p*-value**	**R^2**	**OR [95% CI]**	***p*-value**
Exceeding baseline for MMA[g]	0.073	0.970 [0.870, 1.081]	0.970	0.068	1.002 [0.935, 1.075]	0.949	0.138	0.974 [0.895, 1.059]	0.573
Non diabetic subjects	0.089	0.938 [0.845, 1.041]	0.226	0.090	1.022 [0.960, 1.088]	0.489	0.095	0.984 [0.917, 1.055]	0.984
Adjusted for total arsenic	0.077	0.985 [0.894, 1.084]	0.754	0.109	1.011 [0.950, 1.077]	0.724	0.119	0.971 [0.904, 1.042]	0.411
Adjusted for arsenobetaine[h]	0.073	0.972 [0.882, 1.070]	0.558	0.125	1.008 [0.945, 1.075]	0.809	0.112	0.980 [0.913, 1.051]	0.570
Adjusted for creatinine[i]	0.075	0.976 [0.890, 1.070]	0.600	0.118	1.024 [0.962, 1.090]	0.456	0.113	0.981 [0.914, 1.052]	0.590

[a] adjusted for age (continuous), gender, race (dichotomous, White vs. non-White) smoking status (dichotomous), and poverty status (PIR<1 vs. PIR≥1); [b] Urine % MMA; [c] BMI<25.0; [d] 25.0 ≥BMI<30.0; [e] BMI≥30.0; R^2 represents Cox & Snell R^2 value; [f] *p*-value obtained from binary logistic regression (* significant at (α = 0.05)); [g] urine MMA ≥0.20μg/L; [h] arsenic in seafood; [i] measure of hydration

Table S4

Subgroup and sensitivity analyses in fully adjusted[a] binary logistic regression model of men

IV: SMI[b]

DV: Metabolic Syndrome

	Normal BMI[c]			Overweight BMI[d]			Obese BMI[e]		
**Additional Criteria**	**R^2**	**OR [95% CI]**	***p*-value[f]**	**R^2**	**OR [95% CI]**	***p*-value**	**R^2**	**OR [95% CI]**	***p*-value**
Exceeding baseline for MMA and DMA[f]	0.117	06.069 [0.296, 124.56]	0.242	0.069	0.901 [0.176, 4.609]	0.900	0.134	0.768 [0.092, 6.375]	0.807
Non diabetic subjects	0.085	2.356 [0.361, 15.367]	0.370	0.095	0.447 [0.118, 1.695]	0.236	0.094	0.845 [0.249, 2.872]	0.787
Adjusted for total arsenic	0.077	1.125 [0.162, 7.786]	0.905	0.111	0.587 [0.151, 2.285]	0.443	0.116	1.367 [0.329, 5.682]	0.666
Adjusted for arsenobetaine[g]	0.072	1.551 [0.224, 10.738]	0.656	0.127	0.624 [1.153, 2.540]	0.511	0.110	0.895 [0.256, 3.127]	0.862
Adjusted for creatinine[h]	0.074	1.414 [0.228, 8.764]	0.710	0.122	0.446 [0.117, 1.695]	0.236	0.111	0.887 [0.260, 3.029]	0.848

[a] adjusted for age (continuous), gender, race (dichotomous, White vs. non-White) smoking status (dichotomous), and poverty status (PIR<1 vs. PIR≥1); [b] Secondary methylation index; [c] BMI<25.0; [d] 25.0 ≥BMI<30.0; [e] BMI≥30.0; R^2 represents Cox & Snell R^2 value; [f] *p*-value obtained from binary logistic regression (* significant at (α = 0.05)); [g] urine DMA ≥1.91μg/L, urine MMA ≥0.20μg/L; [h] arsenic in seafood; [i] measure of hydration
